# CANA v1.0.0: efficient quantification of canalization in automata networks

**DOI:** 10.1093/bioinformatics/btaf461

**Published:** 2025-08-23

**Authors:** Austin M Marcus, Jordan Rozum, Herbert Sizek, Luis M Rocha

**Affiliations:** Center for Complex Biological Systems, University of California Irvine, Irvine, CA 92697, United States; School of Systems Science and Industrial Engineering, Binghamton University (State University of New York), Vestal, NY 13850, United States; School of Systems Science and Industrial Engineering, Binghamton University (State University of New York), Vestal, NY 13850, United States; Department of Informatics, Indiana University, Bloomington, IN 47405, United States; School of Systems Science and Industrial Engineering, Binghamton University (State University of New York), Vestal, NY 13850, United States; Universidade Católica Portuguesa, Católica Biomedical Research Centre, 1649-023 Lisboa, Portugal

## Abstract

**Summary:**

The biomolecular networks underpinning cell function exhibit canalization, or the buffering of fluctuations required to function in a noisy environment. We present a new major release of CANA, v1.0.0, an open-source Python package for understanding canalization in automata network models, discrete dynamical systems in which activation of biomolecular entities (e.g. transcription of genes) is modeled as the activity of coupled automata. One understudied putative mechanism for canalization is the functional equivalence of biomolecular regulators (e.g. among the transcription factors for a gene). We study this mechanism using the theory of symmetry in discrete functions. We present a new exact method, schematodes, for finding maximal symmetry groups among the inputs to discrete functions, and integrate it into CANA. The schematodes method substantially outperforms the inexact method of previous CANA versions both in speed and accuracy. We apply CANA v1.0.0 to study symmetry in 74 experimentally supported automata network models from the Cell Collective (CC) repository. The symmetry distribution is significantly different in the CC than in random automata with the same in-degree (connectivity) and bias (average output) (Kolmogorov–Smirnov test, *P* ≪ .001). Its spread is much wider than in a null model (IQR 0.31 versus IQR 0.20 with equal medians), demonstrating that the CC is enriched in functions with extreme symmetry or asymmetry.

**Availability and implementation:**

CANA source is on https://github.com/CASCI-lab/CANA and is installable via pip install cana. Source for schematodes is on https://github.com/CASCI-lab/schematodes. Analysis scripts are on https://github.com/CASCI-lab/symmetryInCellCollective.

## 1 Introduction

Automata networks are discrete dynamical systems that are popular in systems biology where threshold behaviors are common and data required to fit detailed mechanistic models are rare. They may consider multi-state variables [e.g. proportion of cells undergoing apoptosis in cell cultures; Gómez Tejeda Zañudo *et al.* (2017)], but in simulation and analysis, they are usually converted to isomorphic Boolean networks (BN) whose automata can only take two logical states. Here, we discuss the open-source CANA Python library for BN analysis, with an emphasis on understanding symmetry and redundancy. We present CANA v1.0.0, a substantial upgrade to CANA v0.1.2 (Correia *et al.* 2018, Gates *et al.* 2021). We also introduce schematodes, a new Python library written in Rust that provides the symmetry computations in CANA v1.0.0. In addition to large speed improvements, CANA v1.0.0 includes new functionality for the computation of perturbation response, interaction graphs, and symmetry properties (see Box 1). We redesigned the functions for describing the effect of permuting a Boolean function’s non-redundant inputs using using Cython and Rust PyO3 bindings and a faster, exact algorithm. Here, we focus primarily on this improved symmetry computation.

In biomolecular regulation, canalization (the buffering of genetic, epigenetic, and environmental fluctuations) plays a key role in establishing a robust mapping from genotype to phenotype (Waddington 1942). Robustness of sensors to fluctuations, a feature of canalization, requires dynamical redundancy. This manifests in several ways, including: (i) multiple signaling pathways, (ii) multiple combinations of transcription factors that bind a gene’s promoter region, and (iii) threshold behaviors that allow depletion of one signal to be overcome by overabundance of another. To study canalization, CANA provides routines that quantify various types of redundancy using rigorous measures. It extends the McCluskey (1956) theory of total symmetry of Boolean functions, by compressing the prime implicants of a Boolean function into a set of *schemata*, where symmetry is described with symbols for groups of inputs that can permute (Marques-Pita and Rocha 2013). CANA was first released in 2018 as v0.0.2-alpha by Correia *et al.* (2018) and updated to v0.1.2 by Gates *et al.* (2021). Since then, we have made significant improvements and additions, culminating in CANA v1.0.0.

Box 1: CANA key features (more in Supporting Materials)

**Schema redescription:** Compress redundancy to describe and visualize the causal logic of automata rules parsimoniously.

**Canalization:** Measures of input redundancy and symmetry as parameters of Boolean automata.

**Effective graph:** A probabilistic causal graph with the likely dynamical pathways within a BN.

**Dynamics canalizing map:** An exact causal graph represented as a threshold network with only the state transitions necessary and sufficient to recover BN dynamics.

**Synchronous dynamics:** Routines for BN simulation and control.

**Figure 1.**
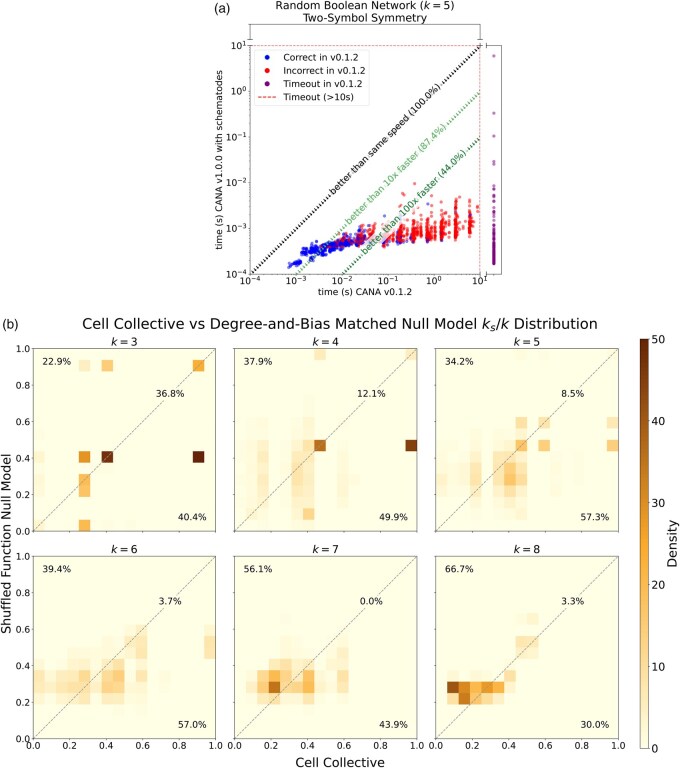
Benchmarks and symmetry analysis for random and CC automata. (a) Computation time using CANA v1.0.0 with schematodes (vertical) and CANA v0.1.2 (horizontal). All outputs from CANA v1.0.0 with schematodes were verified to be correct. Correct (incorrect) outputs generated using the heuristic method of CANA v0.1.2 are shown in blue (red). Benchmarks for the CC are provided in Supplementary Material. Benchmarks were run on a 3.9 GHz Intel core i5 CPU. (b) comparisons of normalized symmetry ($k_{s}/k$) for CC functions before (horizontal axes) and after (vertical axes) random output shuffling, shown separately for each k∈{3,…,8}. Each CC automaton is represented 12 times and is used to produce a “shuffled” null ensemble with equal bias *p* and in-degree *k*. Percentages in the top left, top right, and bottom right of each panel indicate how often shuffles result in increased, equal, or decreased symmetry, respectively.

Here, we also provide a group-theoretic grounding for the symmetry schema redescription of Marques-Pita and Rocha (2013) and develop a new algorithm, schematodes, for prime implicant compression. It is implemented as a Python library written in Rust with PyO3 bindings and is integrated into CANA v1.0.0. While the CANA v0.1.2 permutation symmetry calculation extrapolates from a sample of permutations and can overestimate input permutability, the schematodes algorithm is exact. Despite its exactness, it is dramatically faster than the previous method (Fig. 1a). We demonstrate the use of CANA v1.0.0 with schematodes to study symmetry in biomolecular network models using the 74 experimentally supported Boolean models available in the Cell Collective (CC) (Helikar *et al.* 2012) as a test-bed. We discover that the CC models exhibit more extreme symmetry and asymmetry than random models that control for number of inputs (in-degree, *k*) and average output (bias, ρ). In other words, moderately symmetric functions are underrepresented in the CC.

## 2 Two-symbol schemata theory

In this section, we summarize the theory of two-symbol schemata of Marques-Pita and Rocha (2013) for compressing the Boolean lookup tables (LUTs) that map input configurations (the $2^{k}$ possible values of *k* input nodes) to output values. Formal details are in Supporting Materials. Biologically, the compression achievable (by removing redundancy) is related to the extent of regulatory functional equivalence, which is relevant in phenomena such as genome duplication, compensatory mutation, and drug resistance (Marques-Pita and Rocha 2013, Gates *et al.* 2021).

CANA uses the prime implicants (minimal subsets of activating inputs) of a Boolean function and its negation to construct *one-symbol schemata*. The input configurations leading to each output state are grouped and compressed into schemata composed from the set of symbols 1, 0, and #, representing ON, OFF, and “don’t care.” For example, the two-input OR function, $f(x)=x_{1}\vee x_{2}$ has prime implicants 1# and #1, and its negation, the AND function $\neg f(x)=\neg x_{1}\wedge \neg x_{2}$ has prime implicant 00. So, the LUT of *f*, {00}↦0, {01,10,11}↦1, can be compressed to the set of one-symbol schemata: {1#,#1}↦1, {00}↦0, which shows that one of the inputs is *redundant* if the other is set to 1. This schemata compression allows CANA to compute various measures of micro-level canalization, such as effective connectivity (a measure of input redundancy), as well as causal maps of macro-level dynamics such as the effective graph (Gates *et al.* 2021).

One-symbol schemata capture redundancy of input values, but considering position redundancy (i.e. permutability of inputs) allows further compression. CANA introduces a second set of symbols (°, ^, etc.) to indicate which one-symbol schemata inputs can permute without affecting the output state. These annotated schemata are called *two-symbol schemata*. For instance, the one-symbol schemata {1#,#1}↦1 are further compressed into a two-symbol schema $\{\overset{{}^{\circ}}{1}\overset{{}^{\circ}}{\#}\}\mapsto 1$. Not all inputs arbitrarily permute in general, so we identify *partial symmetries*, maximal permutation symmetries of input subsets (e.g. $\overset{{}^{\circ}}{1}\overset{{}^{\circ}}{\#}0$ represents permutations of only the fist two inputs). Multiple such permutations can coexist, as in $\overset{{}^{\circ}}{\#1}\overset{{}^{\circ}}{0}\hat{0}\hat{1}$, a two-symbol schema of $f(x)=x_{2}\wedge (\neg x_{1}\vee \neg x_{3})\wedge (x_{4}\wedge \neg x_{5}\vee \neg x_{4}\wedge x_{5})$. It compresses the set of one-symbol schemata {#1001,#1010,01#01,01#10}, which in turn compresses the following subset of LUT entries of *f* : {01001,01010,01001,01010,11001,11010,01101,01110}.

We disregard trivial permutations of identical symbols such as swapping the inputs in {00}↦0, as this provides no additional information for control interventions. Instead, we seek permutations that do not map each input configuration to itself; formally, these form *faithful* group actions as discussed in the Supporting Materials. We identify the maximal sets of input configurations that are invariant under such permutations using a new exact algorithm implemented in Rust as a Python package schematodes, which is integrated into CANA v1.0.0. See Supporting Materials for a formal description of two-symbol symmetry and pseudo-code for the schematodes algorithm. While we are motivated by permutations within a 3-symbol alphabet (#, 0, 1), schematodes extends to arbitrary alphabets, allowing for future analyses of multi-valued logic.

CANA calculates various quantities and representations derived from two-symbol schemata. For instance, the dynamic canalization map (DCM), introduced by Marques-Pita and Rocha (2013), represents a BN as a threshold network with the necessary and sufficient control logic revealed after redundancy removal. By leveraging permutation symmetry, the DCM is generally more compact than similar representations (Klarner *et al.* 2015). To quantify the amount of partial symmetry in a Boolean function, CANA v1.0.0 implements the $k_{s}$ input-symmetry measure of Marques-Pita and Rocha (2013). The input symmetry, $k_{s}$, is the average number of permutation indices (faithfully permuting inputs) in the two-symbol schemata of *f*, aggregated by input; see Supporting Materials. Alternative aggregations, such as maximum rather than average, are also available. Similarly, by default, CANA identifies faithful symmetries of prime implicants when constructing two-symbol schemata and computing derived measures, but several alternatives are implemented.

## 3 Symmetry in random and cell collective models

Input redundancy and its dual, effective connectivity, have been studied in random and systems biology models (Gates *et al.* 2021, Manicka *et al.* 2022, Costa *et al.* 2023). Here, we focus on the under-studied prevalence of symmetry redundancy in these models. We computed two-symbol schemata and input symmetry, $k_{s}$, for the 3462 functions of 74 BNs from the CC (Helikar *et al.* 2012) and for an ensemble of 1943 randomly generated functions with in-degree k=5 and bias ρ∈[0,1]. Figure 1 (left panel) compares CANA v1.0.0 and CANA v0.1.2 speed and accuracy (computed by exhaustive evaluation) in random automata. The CANA v0.1.2 heuristic algorithm produces incorrect two-symbol schemata in 58 of the unique CC functions (12%) and in 53% of the randomly generated test ensemble. The CANA v1.0.0 schematodes algorithm is not heuristic, and is 100% accurate. It completed all computations in the random ensemble in under 10 s, and its speed is greatly improved for most functions (≥  10x faster in 87% and ≥  100x in 44% of random k=5 functions tested). Functions with ≤  10x speed improvement are generally highly symmetric.

Most CC functions (98%) are invariant under permutation of an essential input (a 0 or a 1 whose bit-flip changes the output) with a wildcard (a # whose bit-flip does not change the output), highlighting collective or group-constrained redundancy, which goes beyond simple bit-flipping (Marques-Pita and Rocha 2013). One way such patterns can arise is from combining variables by nesting only AND (∧) or OR (∨) operators, which can be viewed as equal-weight threshold functions with the largest (*k*) or smallest (1) threshold, respectively. For example, $x_{1}\vee x_{2}\vee x_{3}$ is a threshold function with threshold 1 and two-symbol schema $\overset{{}^{\circ}}{1}\overset{{}^{\circ}}{\#}\overset{{}^{\circ}}{\#}\mapsto 1$ and 000↦0. Thresholds between 1 and *k* result in permutations of more than just a single 0 or 1 with wildcards; e.g. $\left(x_{1}\wedge x_{2})\vee (x_{2}\wedge x_{3})\vee (x_{1}\wedge x_{3}\right)$ with threshold 2 has two-symbol schemata $\overset{{}^{\circ}}{1}\overset{{}^{\circ}}{1}\overset{{}^{\circ}}{\#}\mapsto 1$ and $\overset{{}^{\circ}}{0}\overset{{}^{\circ}}{0}\overset{{}^{\circ}}{\#}\mapsto 0$. While only 2% (11) of CC functions have two-symbol schema that permute a 0 with a 1, two thirds of the functions in our random ensemble have this property. This is consistent with the over-representation in the CC of monotonic (unate) functions—those with the property that every input is unambiguously an activator or an inhibitor. Using CANA v1.0.0, we discovered that >90% of CC models contain only monotonic functions (see Supporting Materials).

To assess whether the symmetry distribution observed in the CC is expected from the number of regulators (*k*) and bias (ρ) alone, we shuffled the output column of the LUTs of each CC function to produce twelve random rules (preserving the original *k* and ρ) thereby generating a degree-and-bias matched null model. We then compared the normalized symmetry, $k_{s}/k$, of the original CC functions with that of their shuffled sets (Fig. 1, right panels). For k≤6, shuffling tends to decrease symmetry, whereas the opposite occurs for larger in-degree. Furthermore, we observe a larger spread in $k_{s}/k$ values for the CC functions relative to the null model: IQR 0.31 versus IQR 0.20 for 3≤k≤8 with median value 0.375 for both (distributions are significantly different by the Kolmogorov–Smirnov test at *P* ≪ .001). Highly symmetric functions are overrepresented in the CC when *k* is low; e.g. for k=3, the 75th percentile of $k_{s}/k$ is 0.88 for CC functions, but only 0.42 when shuffled. Some intermediate values of $k_{s}/k$ are absent in the CC but appear in the shuffled functions (vertical light bands in Fig. 1, e.g. near $k_{s}/k=0.3$ for k=4,5).

## 4 Discussion

CANA enables analysis of canalization in automata, providing valuable insight into the role of redundancy in the robustness and control of biological networks. Previous research emphasized measures of canalization derived from prime implicants (Gates *et al.* 2021, Costa *et al.* 2023), monotonicity (Grefenstette *et al.* 2006), or single node perturbations (Shmulevich and Kauffman 2004). Yet, symmetry of regulatory functions is understudied despite work suggesting it is an important canalization mechanism in biological regulation (Reichhardt and Bassler 2007, Kadelka *et al.* 2024). For example, Grefenstette *et al.* (2006) show how increased symmetry arises from the interactions of biological signaling components with binding domain duplication and modification. Models of genome duplication in yeast (Conant and Wolfe 2006, Anholt and Mackay 2023) suggest that symmetry among duplicated elements is only lost over very long timescales. Symmetry in regulatory functions may also help explain the robustness of cell systems to perturbations observed by Park *et al.* (2023). Exploring these concepts requires careful quantification of symmetry in computational models. We therefore developed a formal foundation for symmetry measures previously used in automata networks. We built upon this foundation to create the open-source Python package CANA v1.0.0 and its novel component schematodes, which we applied to study symmetry in biomolecular networks.

Using CANA, we found that the symmetry parameter $k_{s}/k$ has a wider distribution (IQR 50% larger) in the experimentally supported CC models than expected by chance, demonstrating that symmetry in models of biological regulation is not random. Our analysis of two-symbol schemata in the CC is consistent with the frequently observed tendency toward monotonicity in regulatory functions and demonstrate a preference for highly symmetric threshold-like functions. Uncovering the role of related features, such as the prevalence of nested canalizing functions (Kadelka *et al.* 2024) remains for future work.

The symmetry calculations in CANA v1.0.0 are much faster than in CANA v0.1.2. They are now exact and generalized to more than two states. The one- and two-symbol schema computations available in CANA v1.0.0 are essential for a full characterization of canalization in biological networks. Thus, CANA v1.0.0 presents an opportunity to provide new explanations for the robust functioning of biochemical regulation.

## Supplementary Material

btaf461_Supplementary_Data

## Data Availability

CANA source is on https://github.com/CASCI-lab/CANA and is installable via pip install cana. Source for schematodes is on https://github.com/CASCI-lab/schematodes. Analysis scripts are on https://github.com/CASCI-lab/symmetryInCellCollective.
