# Supplementary material for: CANA v1.0.0: efficient quantification of canalization in automata networks
Source: Bioinformatics. 2025 Aug 23;41(10):btaf461. doi: 10.1093/bioinformatics/btaf461 (PMC12512137; doi:10.1093/bioinformatics/btaf461)
Supplement: btaf461_Supplementary_Data [file btaf461_supplementary_data.pdf]

Supporting Material

AM Marcus, JC Rozum, H Sizek, LM Rocha

August 19, 2025

1 Overview of Other CANA Features

`CANA` v0.1.2 was released in 2018, and its features were described in Correia et al. (2018). In the main text, we have focused on new contributions in the area of Boolean symmetry, but `CANA` also has many tools for attractor analysis, control, redundancy computations, and network construction that were provided in the 2018 release or have been added in the intervening years. In addition to new features, we have incorporated more rigorous unit-testing and continuous integration into the `CANA` development workflow, incorporated multiple bugfixes and optimizations, and improved readability of the code and documentation. In this section, we briefly describe some of the features implemented in `CANA` that were not emphasized in the main text.

Attractor detection, simulation, and control. `CANA` provides an interface to the BNS attractor-detection tool for finding the attractors of a Boolean network under asynchronous update (Dubrova and Teslenko, 2011), which uses an efficient SAT-based method. We also implement a feedback vertex set-based attractor control algorithm. `CANA` additionally provides basic brute-force algorithms for building the state transition graph, determining the fate of initial conditions, and testing the effects of node-level interventions. These brute-force methods are implemented for synchronous update. Though not implemented in `CANA` directly, we have previously presented `cubewalkers` (Park et al., 2023), which is interoperable with `CANA` and allows for GPU-accelerated simulation of network dynamics under various update schemes. Moreover, it allows for computation of various dynamical quantities, discussed in Park et al. (2023).

Redundancy measures and related concepts. In addition to the symmetry measures that are the focus of this work, `CANA` provides tools for computing and analyzing prime implicants of Boolean functions. We have previously presented the concepts of effective connectivity and edge effectiveness Marques-Pita and Rocha (2013); Gates et al. (2021), which can be computed in `CANA`. Effective connectivity is a measure of collective canalization that is computed as the average size of the subset of inputs necessary to determine the output of a Boolean function. In Gates et al. (2021), a method was proposed to apportion effective connectivity among the incoming edges of a Boolean node, resulting in the edge-level measure of edge effectiveness. `CANA` also includes methods for

computing the sensitivity parameter of Shmulevich and Kauffman (2004). As discussed in the main text, `CANA` computes redundancy measures using a schema redescription framework. Previously, this was accomplished using a heuristic algorithm, but this has since been replaced by `schematodes`. `CANA` has methods for visualizing these schema as tables, which have previously been described in Correia et al. (2018).

Network construction and data sets. We have included in `CANA` a library of 74 experimentally-supported models of cell processes from the Cell Collective (Helikar et al., 2012). In addition, `CANA` includes methods for generating random Boolean networks of various kinds. `CANA` is able to construct the interaction graph between Boolean nodes as a `networkx` object. The effective graph (Gates et al., 2021) is an edge-weighted interaction network in which edge weights are determined by edge effectiveness. It was shown to capture key dynamical information related to information spread and redundancy in Boolean networks. The dynamics canalizing map (DCM) was introduced as a way to structurally represent Boolean update functions as a threshold network (Marques-Pita and Rocha, 2013), similar to hypergraph representations used by Wang and Albert (2011); Klarner et al. (2015); Rozum et al. (2021) and others. In contrast to these other approaches, which encode only the prime implicants, the DCM additionally encodes information about the two-symbol schemata discussed in the main text. See Marques-Pita and Rocha (2013) for details. `CANA` includes functions for constructing and plotting the DCM of a Boolean network.

2 Formal description of two-symbol symmetry

In this section, we develop the formal definition of two-symbol schemata, beginning with definitions from introductory group theory. Readers who are familiar with the basics of group theory and group actions may wish to skip ahead to Definition 8 (symmetric group of an index subset).

Definition 1 (group) *A group G is a nonempty set with an associative binary operation $* : G \times G \to G$ such that*

- *there is an identity element $e \in G$ satisfying $e * g = g * e = g$ for all $g \in G$ and*
- *every $g \in G$ has an inverse element $g^{-1} \in G$ satisfying $g * g^{-1} = g^{-1} * g = e$.*

For the sake of brevity, the $*$ symbol can be omitted from group products when its presence can be inferred from context, e.g., $g * h$ can be written gh. Note that, in general, gh need not equal hg. We will be primarily concerned with smaller groups that lie within a larger group of transformations, and so we recall the definition of a subgroup.

Definition 2 (subgroup) *A subset $H \subseteq G$ is a subgroup of G if H forms a group under the restriction of $*$ to H.*

There are various operations that one can perform on groups and their subsets. One operation that is particularly important for understanding partial symmetry is the product of group subsets, which is the set formed by taking all (ordered) group products of elements from two or more sets.

Definition 3 (product of group subsets) *If S and T are subsets of G, the product ST is the set $ST = \{st : s \in S \text{ and } t \in T\}$.*

Note that ST is not necessarily a subgroup of G. If, however, S and T are subgroups of G that commute ($ST = TS$) and have a trivial intersection ($S \cap T = \{e\}$), then ST is a subgroup of G because $*$ acts on the two terms of the product st independently, i.e., $s_0 t_0 s_1 t_1 = s_2 t_2$ where $s_2 = s_0 s_1$ and $t_2 = t_0 t_1$. This is a sufficient, but not necessary, condition for ST to be a subgroup of G.

We consider how a group G affects elements of some set of objects X through the lens of (left) group actions.

Definition 4 (left group action) *A left group action of a group G on a set X is a function $\alpha : G \times X \to X$ such that for all $g, h \in G$ and $x \in X$*

- *$\alpha(e, x) = x$ and*
- *$\alpha(g, \alpha(h, x)) = \alpha(g * h, x)$.*

For the sake of brevity, when α is clear from context, $\alpha(g, x)$ can be written as $g \cdot x$ or gx. All group actions discussed here are left group actions (as opposed to right group actions); thus we simply refer to left group actions as group actions. To characterize the group actions that are relevant for two-symbol schemata, we recall two properties, transitivity and faithfulness, that distinguish different types of group actions.

Definition 5 (transitive group action) *A group action of G on X is transitive if for all $x, y \in X$, there exists a $g \in G$ such that $gx = y$.*

Definition 6 (faithful group action) *A group action of G on X is faithful if $gx = x$ for all $x \in X$ implies $g = e$ is the identity element of G.*

Transitivity formalizes the concept of being able to reach all elements of a set by application of the group action, regardless of where in the set one begins. Faithfulness formalizes the concept of a group action specifying a nontrivial effect for all elements of the group. We must also consider the effect a (sub)group action has on a subset of X.

Definition 7 (restriction of a group action) *A group action α of G on X restricts to a subgroup H of G and a subset Y of X if $\alpha(h, y) \in Y$ for all $h \in H$ and all $y \in Y$.*

Two-symbol symmetry is concerned with the effects of permuting inputs to a function. Thus, we recall the definition of a symmetric (permutation) group on a set of indices.

Definition 8 (symmetric group of an index subset) *The symmetric group of an index subset $m \subseteq \{1, \ldots, k\}$, denoted S_m, is the set of permutations of m with the group operation of composition.*

We use two-cycle notation to denote elements of S_m; the pair (a, b) indicates that the ath and b^{th} elements are interchanged under the permutation. All permutations are expressible as compositions of two-element swaps. Any symmetric group of an index set has a natural group action on the set X^k (the set of k-tuples with entries drawn from X) in which $g \in S_m$ acts on a tuple $x \in X$ by permuting the entries of x in the indices indicated by g. That is, $g = (a, b)$ acts on $x = x_1 \ldots x_k$ by swapping x_a and x_b. The action of all other permutations follows from composing transpositions. Note that if some symbols in x are the same, some elements of g will act on x by mapping x to itself. Recall that if any x is held fixed by all elements of S_m, then the natural action of S_m on X^k is not faithful. The natural action of S_m will not generally restrict to a proper subset Y of X^k, so we are motivated to consider special subgroups of S_m that may restrict to Y while retaining a flavor of arbitrary permutation.

Definition 9 (partially symmetric group) *A partially symmetric group is a product of symmetric groups $S_{m_1} \ldots S_{m_n}$ where each pair of index sets m_i and m_j are disjoint.*

Note that because the index sets are disjoint, and because each S_{m_i} is a subgroup of $S_{\{1,\ldots,k\}}$, every partially symmetric group is indeed a group. Furthermore, every partially symmetric group has a similar natural group action to that of symmetric groups that is obtained by restricting the domain of the natural group action on $S_{\{1,\ldots,k\}}$ to $S_{m_1} \ldots S_{m_n}$. Furthermore, because the index sets are disjoint, their ordering has no meaning, and $S_{m_1} S_{m_2} = S_{m_2} S_{m_1}$, for example.

Definition 10 (two-symbol schema) *A two-symbol schema of a set $F \subseteq X^k$ is a pair $(Z, S_{m_1} \ldots S_{m_n})$, where Z is a subset of F and $S_{m_1} \ldots S_{m_n}$ and a partially symmetric group satisfying the following properties:*

1. *the natural group action of $S_{m_1} \ldots S_{m_n}$ restricts to a faithful and transitive group action on Z, and*
2. *Z is inclusion maximal.*

Here condition 2 means that, if there exists $\left(Z', S_{m'_1} \ldots S_{m'_{n'}}\right)$ satisfying property 1, then Z is not a proper subset of Z'.

For the sake of brevity, we express $(Z, S_{m_1} \ldots S_{m_n})$ using a representative $z \in Z$ and indicate each index set m_i by a distinct symbols, or decorators, above the indices m_i contains. For example,

$$\mathring{1}0\mathring{2}\hat{2}\hat{3} \tag{1}$$

is short-hand for

$$\left(\{10223, 10232, 20123, 20132\}, S_{\{0,2\}}S_{\{3,4\}}\right). \tag{2}$$

The "two" in "two-symbol schema" refers to the fact that this short-hand uses two symbol sets: the set X, and the set of decorator symbols.

In the context of a Boolean function, f, we set k equal to the number of inputs to that function. We can consider X to be the set $\{0,1\}$, in which case we are interested in the two-symbol schemata of the sets $F_0 = \left\{x \in X^k : f(x) = 0\right\}$ and of $F_1 = \left\{x \in X^k : f(x) = 1\right\}$. More commonly, however, we consider $X = \{0, 1, \#\}$, where the # symbol denotes a "wild-card" or unspecified variable value, and we find the two symbol schemata of the subsets F_1' and F_0' of X^k, which denote the set of all prime implicants of f and its negation, respectively. We denote the set of all two-symbol schemata for a Boolean function f as $TSS(f)$, and denote $TSS(f,x)$ the subset of $TSS(f)$ for which each Z_i contains x. Specifically, we write $TSS(f,x) = \left\{\left(Z_i, S_{m_{i,1}} \dots S_{m_{i,n}}\right) \in TSS(f) : x \in Z_i\right\}$.

Definition 11 (LUT Input Symmetry) *The LUT input symmetry of an input $x \in \{0,1\}^k$ to a Boolean function f, denoted $sym(f,x)$, is the average number of summed permutation indices in $TSS(f,x)$. That is,*

$$sym(f,x) = \sum_{\left(Z, S_{m_1} \dots S_{m_n}\right) \in TSS(f,x)} \frac{|m_i|}{|TSS(f,x)|}. \tag{3}$$

Definition 12 (Input Symmetry k_s; Marques-Pita and Rocha (2013)) *The input symmetry measure k_s of a Boolean function f is the LUT input symmetry of its inputs:*

$$k_s = \frac{1}{2^k} \sum_{x \in \{0,1\}^k} sym(f,x). \tag{4}$$

In other words, k_s is the average number of permutation indices in the two-symbol schemata of f, aggregated by input.

3 Schematodes algorithm

The main algorithm for schematodes is provided in this section. We break the main algorithm into two pieces so as to aid interpretation.

Algorithm 1 Main algorithm of schematodes.

Require: *tuples*, a set of tuples of equal length
Ensure: *tss*, a set of two symbol symmetry schema

function SCHEMATODES(*tuples*)
  $tss \leftarrow \emptyset$
  $sig \leftarrow$ subsets of *tuples* grouped by signature
  for $oss \in sig$ **do**
    $tss \leftarrow tss \cup$ TWOSYMBOLS(oss)
  return tss

Algorithm 2 Two symbol symmetry calculation for sets of tuples with equal counts of each unique entry.

Require: *oss*, set of equal-length tuples with equal signature
Ensure: *sym*, a set of two symbol symmetry schema

function TWOSYMBOLS(*oss*)
  $sym \leftarrow \emptyset$
  $seen \leftarrow \emptyset$
  $k \leftarrow \mathbf{len}(oss)$
  $tc \leftarrow \{i \in 1..k : \forall t_1, t_2 \in oss, t_1[n] = t_2[n]\}$
  $T_f \leftarrow \{(i,j) \in S_{\{1,\ldots,k\}} : i, j \notin tc\}$ ▷ *potentially faithful transpositions*
  for $z_0 \in oss$ **do**
    $candidates \leftarrow \{(i,j) \in T_f : (i,j) \cdot z_0 \in oss\}$
    $\mathcal{C} \leftarrow$ power set of *candidates*, partially ordered in decreasing size
    for $S \in \mathcal{C}$ **do**
      if $\exists S' \in seen : S \subseteq S'$ **then**
        continue;
      $Z \leftarrow \{z_0\}$
      repeat
        $size \leftarrow |Z|$
        $Z \leftarrow \{s \cdot z : s \in S, z \in Z\}$
      until $size = |Z|$
      if $Z \nsubseteq oss$ **then** ▷ *check for closure*
        continue;
      if $\exists s \in S \neq e : \forall z \in Z, s \cdot z = z$ **then** ▷ *check for faithfulness*
        continue;
      $seen \leftarrow seen \cup \{S\}$
      $G \leftarrow \langle S \rangle$ ▷ *get group generated by S*
      $sym \leftarrow sym \cup \{(Z, G)\}$
  return sym

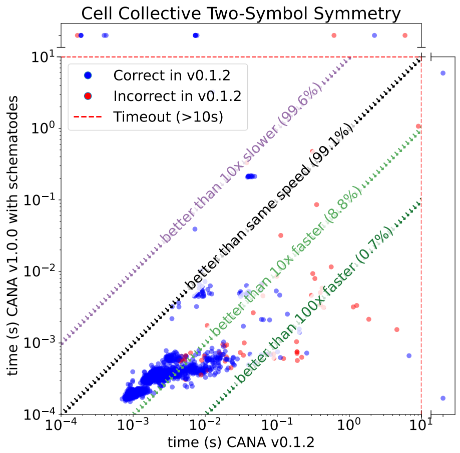

Figure 1: Benchmarks for random automata and symmetry analysis for random and CC automata. **a)** computation time using `CANA` v1.0.0 with `schematodes` (vertical) and `CANA` v0.1.2 (horizontal). All outputs from `CANA` v1.0.0 with `schematodes` were verified to be correct. Correct (incorrect) outputs generated using the heuristic method of `CANA` v0.1.2 are shown in blue (red). Benchmarks were run on a 3.9GHz Intel core i5 CPU.

4 Additional Benchmark

In addition to the random function benchmark presented in the main text, we also conducted a smaller benchmark on functions that appear in the Cell Collective Helikar et al. (2012). The results are presented in this supplement, in Figure 1. We find a general increase in performance, though not as dramatic as in random functions. `CANA` v1.0.0 is improved in over 99% of cases, and is faster than 10x in 8.8% of cases. `CANA` v1.0.0 produces the correct output in all completed runs, whereas the heuristic algorithm of `CANA` v0.1.2 produces incorrect output in some cases (shown with red dots in Figure 1).

5 Other remarks and examples

Remark 1 (Two-symbol schemata and monotonicity) *A function that has a two-symbol schema of the form* $\mathring{0}\mathring{1}\dots$ *is not monotonic. This is easily verified by observing that such a function (or its negation) has a prime implicant of the form* $\neg x_1 \wedge x_2 \wedge \dots$ *as well as one of the form* $x_1 \wedge \neg x_2 \wedge \dots$*. Thus, the function's Blake Canonical Form contains both* x_1 *and* $\neg x_1$*, implying it is not monotonic in* x_1*. This observation is sufficient to prove the result, but we also add some intuition: there is at least one input configuration in which toggling* x_1 *from* 0 *to* 1 *changes the output from* 0 *to* 1*, but there is also at least one input configuration in which toggling* x_1 *from* 1 *to* 0 *achieves the same thing. Thus,*

the function is not monotonic in x_1 (or in x_2 by similar reasoning).

Example 1 (Calculation of k_s) *Consider $f(x) = x_1 \vee x_2$. As discussed in the main text, its LUT is $\{00\} \mapsto 0$, $\{01, 10, 11\} \mapsto 1$, with one-symbol schema $\{00\} \mapsto 0$, $\{\#1, 1\#\} \mapsto 1$ and two-symbol schema $\{00\} \mapsto 0$, $\{\mathring{1}\mathring{\#}\} \mapsto 1$. Note that 00 is not annotated by permutation symbols because interchanging the first two inputs of 00 has no effect at the level of one-symbol schemata (it is not a* faithful *action). In contrast, interchanging the first two inputs of 11 does interchange the two one-symbol schemata that correspond to that input configuration (as is also the case with 10 and 01). Thus, on average, each input configuration maps to a two-symbol schema with $(0 \times 1 + 2 \times 3)/4 = 3/2$ permutable inputs and so $k_s = 3/2$.*

Example 2 (Variants of k_s) *Consider $f(x) = x_1 \vee x_2$. We may consider two variants of the symmetry calculations leading to $k_s = 3/2$. In the first, we may relax the requirement that a permutation group action be faithful, in which case we consider the inputs 00 to to be permutable–this leads to a* same symbol *symmetry parameter $k_s^{ss} = 2$. In the second, we can consider symmetry compression on the LUT itself, rather than on the one-symbol schemata. This leads to the representation $\{00\} \mapsto 0$, $\{\mathring{1}\mathring{0}, 11\} \mapsto 1$. Using this representation to compute a symmetry parameter results in $k_s^{\star} = 1$ when requiring faithfulness, or $k_s^{\star ss} = 4$ when allowing same-symbol permutations.*

References

R. B. Correia, A. J. Gates, X. Wang, and L. M. Rocha. CANA: A Python Package for Quantifying Control and Canalization in Boolean Networks. *Frontiers in Physiology*, 9, 2018. ISSN 1664-042X.

E. Dubrova and M. Teslenko. A SAT-Based Algorithm for Finding Attractors in Synchronous Boolean Networks. *IEEE/ACM Transactions on Computational Biology and Bioinformatics*, 8(5), Sept. 2011. doi: 10.1109/TCBB.2010.20.

A. J. Gates, R. Brattig Correia, X. Wang, and L. M. Rocha. The effective graph reveals redundancy, canalization, and control pathways in biochemical regulation and signaling. *Proceedings of the National Academy of Sciences*, 118(12), Mar. 2021. doi: 10.1073/pnas.2022598118.

T. Helikar, B. Kowal, S. McClenathan, M. Bruckner, T. Rowley, A. Madrahimov, B. Wicks, M. Shrestha, K. Limbu, and J. A. Rogers. The Cell Collective: Toward an open and collaborative approach to systems biology. *BMC Systems Biology*, 6(1), Aug. 2012. ISSN 1752-0509. doi: 10.1186/1752-0509-6-96.

H. Klarner, A. Bockmayr, and H. Siebert. Computing maximal and minimal trap spaces of Boolean networks. *Natural Computing*, 14(4), Dec. 2015. doi: 10.1007/s11047-015-9520-7.

M. Marques-Pita and L. M. Rocha. Canalization and Control in Automata Networks: Body Segmentation in Drosophila melanogaster. *PLOS ONE*, 8 (3):e55946, Mar. 2013. doi: 10.1371/journal.pone.0055946. Publisher: Public Library of Science.

K. H. Park, F. X. Costa, L. M. Rocha, R. Albert, and J. C. Rozum. Models of Cell Processes are Far from the Edge of Chaos. *PRX Life*, 1(2), Dec. 2023. doi: 10.1103/PRXLife.1.023009.

J. C. Rozum, J. G. T. Zañudo, X. Gan, D. Deritei, and R. Albert. Parity and time reversal elucidate both decision-making in empirical models and attractor scaling in critical Boolean networks. *Science Advances*, 7(29), July 2021. doi: 10.1126/sciadv.abf8124.

I. Shmulevich and S. A. Kauffman. Activities and sensitivities in boolean network models. *Phys Rev Lett.*, 93(048701), 2004. doi: 10.1103/PhysRevLett.93.048701.

R.-S. Wang and R. Albert. Elementary signaling modes predict the essentiality of signal transduction network components. *BMC Systems Biology*, 5(1), Mar. 2011. doi: 10.1186/1752-0509-5-44.
